# Habitat Loss Shapes Isotopic Niche Responses of a Didelphid Opossum to Fragmentation in Neotropical Semideciduous Dry Forests of Central Brazil

**DOI:** 10.1002/ece3.73558

**Published:** 2026-04-29

**Authors:** Ingrid de Mattos, Juliana Fernandes Ribeiro, Bárbara Zimbres, Gabriela Bielefeld Nardoto, Jader Marinho‐Filho

**Affiliations:** ^1^ Instituto de Ciências Biológicas, Departamento de Zoologia Universidade de Brasília Brasília Brazil; ^2^ Instituto de Ciências Biológicas, Departamento de Ecologia Universidade de Brasília Brasília Brazil; ^3^ Amazon Environmental Research Institute (IPAM) Brasília Brazil

**Keywords:** Cerrado, feeding habits, *Gracilinanus agilis*, habitat amount, stable isotopes

## Abstract

Habitat loss and fragmentation have notable effects on species' trophic ecology, often most pronounced in landscapes with intermediate levels of remaining habitat, where patch configuration can vary significantly. We assessed how habitat fragmentation influences the carbon‐ and nitrogen‐based isotopic niche space of the agile opossum ( *Gracilinanus agilis* ) across landscapes with varying habitat loss in semideciduous dry forests in central Brazil. We found that δ^13^C values did not vary with patch size or habitat loss, suggesting a consistent reliance on forest resources. The opossum exhibited a niche shift only in intermediate and more conserved landscapes, transitioning from insectivory to frugivory in small and large patches, respectively, with a stronger effect in intermediate landscapes. In the most degraded landscape, niche width expanded in smaller patches, likely due to the inclusion of less valuable dietary items in a context of limited food availability and high forager density. Conversely, there was a niche expansion towards larger patches as one moved from intermediate to more conserved landscapes. This shift was attributed to higher resource availability and diversity in areas with better habitat quality and increased forest complexity. There is clear evidence that fragmentation and habitat loss modify the trophic niche of *G. agilis* , potentially hampering its ecological role as a seed disperser, especially in more degraded landscapes. Although the opossum exhibits high adaptability to landscape alterations through trophic plasticity, even small patches might be crucial for their populations in highly fragmented landscapes due to their reliance on forest resources.

## Graphical abstract

Stable isotope analysis indicates that fragmentation and habitat loss in semideciduous dry forests in the Brazilian Cerrado drive trophic niche shifts in *Gracilinanus agilis* , with potential consequences for its role as a seed disperser.

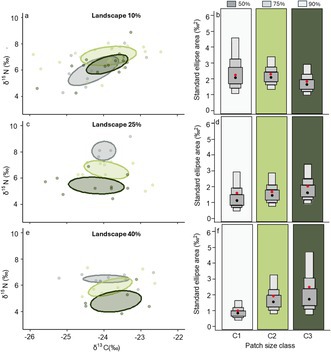

## 1 Introduction

Habitat loss and fragmentation are major drivers of global biodiversity decline, and they are also known to affect trophic relationships (Estes et al. 2011; Haddad et al. 2015). In the tropics, habitat changes primarily result from forest conversion to agriculture and pasture (Fearnside 2001; Gibbs et al. 2010; Strassburg et al. 2017). Although habitat loss has significant negative impacts on ecosystems, fragmentation per se can benefit generalist and tolerant species while jeopardizing specialist ones (Fahrig 2019; Pardini et al. 2010; Hanski 2015). However, the impacts of fragmentation may depend on the interaction between the amount of remaining habitat in the landscape and habitat configuration, and are expected to be strongest at intermediate levels of habitat availability, where patch size and connectivity play a greater role in influencing animal movement across the landscape and, consequently, species persistence (Pardini et al. 2010; Villard and Metzger 2014). Additionally, tolerance to fragmentation may either restrict or expand the range of habitat amount needed for a species to occur (Villard and Metzger 2014).

These responses may depend on a species' ecological traits (Purvis et al. 2000; Davies et al. 2004; Haddad et al. 2015) related to matrix tolerance, dispersal ability, diet, and trophic level (Boyle and Smith 2010; Newbold et al. 2012; Keinath et al. 2017; Magioli et al. 2019). Therefore, the consequences of habitat loss and fragmentation may be reflected in changes in a species' trophic niche (Layman, Quattrochi, et al. 2007; Resasco et al. 2018; Muñoz‐Lazo et al. 2019; Paces et al. 2024). A species' ability to survive in human‐modified landscapes might depend, among other factors, on its trophic plasticity, that is, its capacity to adapt by using the available food resources, including switching to new food items (Resasco et al. 2018; Muñoz‐Lazo et al. 2019), and potentially incorporating resources from the matrix (Magioli et al. 2019).

Diet, trophic niche, and habitat use have been increasingly studied from the perspective of stable isotope analyses of carbon (^13^C) and nitrogen (^15^N) (Layman et al. 2012; Magioli et al. 2019; Leighton et al. 2025). Isotopic carbon ratios (δ^13^C) can help track foraging habitat use, as they vary substantially among primary producers with different photosynthetic pathways (C_3_ trees and shrubs in forests; and C_4_ grasses in savannas and grasslands). Isotopic nitrogen ratios (δ^15^N) indicate trophic position, as there is an enrichment of ^15^N relative to ^14^N at each trophic level within a food web or community (Post 2002; Ben‐David and Flaherty 2012). Therefore, stable isotopes are a promising tool for studying the impacts of habitat conversion, fragmentation, and habitat loss on a species' trophic ecology, providing insights into how animals shift their resource use between continuous forests, isolated habitat patches, and the surrounding matrix (Resasco et al. 2018; Magioli et al. 2019; Muñoz‐Lazo et al. 2019; Navarro et al. 2021).

In this study, we investigated how patch size, a measure of fragmentation, alters the trophic ecology of the omnivorous‐insectivorous agile opossum, *Gracilinanus agilis* (Burmeister, 1854), depending on landscape context of habitat loss in semideciduous dry forests in central Brazil. We asked the following questions: (i) do the increase of fragmentation (patch size reduction) and habitat loss (represented by percentage of natural cover in the landscape) lead the agile opossum to incorporate matrix resources (i.e., an increase in δ^13^C values)? (ii) does fragmentation (i.e., patch size) cause a shift in trophic position of the marsupial depending on landscape context of habitat loss (i.e., an increment in δ^15^N values)? (iii) does isotopic niche space (a measure of trophic diversity) change with patch size depending on the landscape context of habitat loss? We expected *G. agilis* to assimilate matrix resources in smaller patches (higher δ^13^C values) and with increased habitat loss (Magioli et al. 2019; Ribeiro et al. 2019), if the opossum is able to forage in the matrix or matrix arthropods can enter patch edges (Pompermaier et al. 2020). We also predicted an overall increase in the trophic position of *G. agilis* with higher fragmentation, due to greater insect consumption in smaller patches compared to more fruit consumption in larger ones, since fragmentation and habitat loss alter forest dynamics and result in a reduction of fruit availability (Terborgh 1992). This relationship is expected to be stronger in intermediate landscapes, followed by more conserved ones. In more conserved landscapes, overall higher fruit availability might exert higher importance to the opossum's diet. In addition, regarding the size of the isotopic niche space, we expected different responses to increased fragmentation, which would likely be more pronounced at intermediate levels of habitat loss: (i) a niche narrowing, if *G. agilis* tracks the reduced abundance and diversity of food resources, as predicted by the species‐area relationship (Lomolino 2001; Layman, Quattrochi, et al. 2007), and the lower availability of fruit in smaller patches (Terborgh 1986, 1992); or (ii) a niche expansion, resulting from trophic plasticity, allowing opossums to exploit lower‐value food items in smaller patches—especially if they are able to forage on matrix resources (Magioli et al. 2019; Ribeiro et al. 2019).

Because habitat loss is related to landscape connectivity (Fahrig 2003, 2013), highly disrupted landscapes offer fewer opportunities for foraging among patches, potentially reducing the diversity of ingested food items and leading to an overall contraction or expansion of niche space across patches of different sizes. Under these conditions, we expected the effect of patch size on niche space to be weak. Conversely, in more conserved landscapes with high connectivity, consumers are likely able to forage across multiple patches, resulting in similar niche space regardless of patch size. Accordingly, we also predicted a weak effect of patch size (positive or negative) on niche space under conditions of high habitat availability. Finally, we expected the strongest effects of patch size on niche space—either positive or negative—to occur in the intermediate landscape, where moderate connectivity might increase the importance of patch size in shaping the foraging behavior of the agile opossum.

## 2 Materials and Methods

### 2.1 Study Area

We conducted the study in the central portion of the Brazilian Cerrado, a biodiversity hotspot composed of a mosaic of vegetation physiognomies, ranging from open grasslands and savannas to forest formations (Ribeiro and Walter 1998). Sampling patches were established in remnants of semideciduous dry forests within private farms in central Goiás State, where these forests once represented the dominant natural vegetation type. However, because they occur on high‐fertility soils, agriculture and pasture expansion have largely replaced the original vegetation cover (Oliveira‐Filho and Ratter 2002). Additionally, a few patches of savanna vegetation (following Ribeiro and Walter 1998) remain in the region, reflecting the Cerrado as a vegetation complex in central Brazil, where distinct physiognomies often occur along a continuum.

Patches were located in three landscapes of ~15,000 ha with different levels of habitat loss (~10%, 25%, and 40% of remaining native cover) in the municipalities of Abadiânia (16°2′51″ S 48°51′44″ W), Jesúpolis (15°57′05″ S 49°22′26″ W), Jaraguá (15°44′31″ S 49°20′6″ W), Ouro Verde de Goiás (16°13′13″ S 49°11′36″ W), Pirenópolis (15°53′06.40″ S 49°10′46.29″ W), and São Francisco de Goiás (15°55′51″ S 49°15′2″ W) in a highly deforested portion of the Goiás State.

**FIGURE 1:**
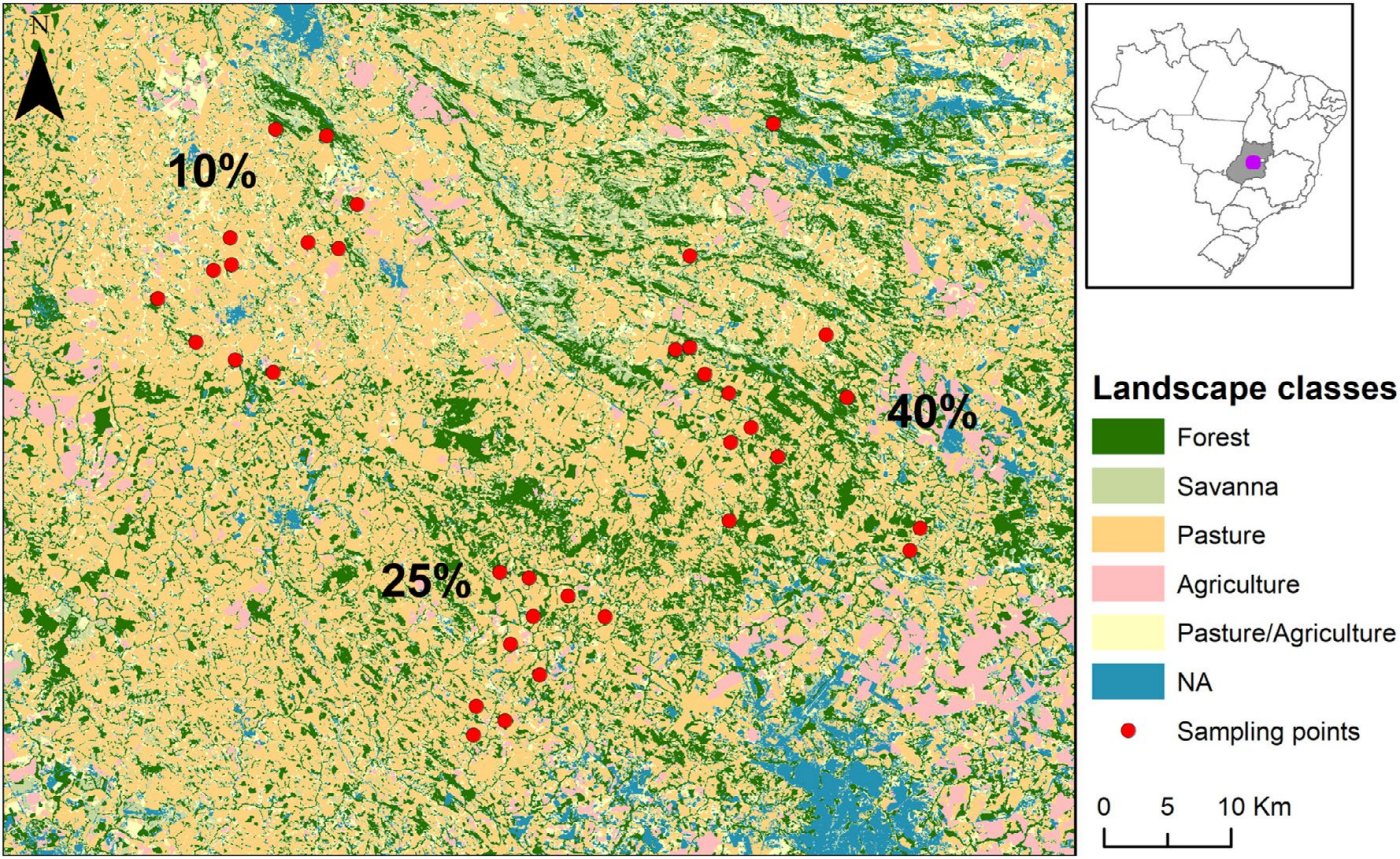
Landscape gradient of habitat loss, estimated from the remaining native cover (considering forest and savanna) of 10%, 25%, and 40% in 15,000‐ha landscapes, showing sampled patches (red dots) in central Goiás State, Brazil. Modified from Mattos et al. (2021).

In each landscape we sampled 12 forest patches (totaling 36 sampling sites), ranging from 2 to 760 ha, where small mammals were surveyed (Figure 1). In this region the matrix is composed mainly of pasture, with a few agricultural areas (such as crop fields and/or banana plantations). The climate is Aw (Köppen), with a hot, wet summer from October to March and a cold, dry winter from April to September.

### 2.2 Studied Species

The agile opossum *Gracilinanus agilis* (Burmeister, 1854) is a didelphid marsupial widely distributed in South America (Voss and Jansa 2009), and in Brazil it inhabits forest formations (Vieira and Palma 2005) in the Cerrado, Caatinga, and Pantanal biomes (Paglia et al. 2012). In the study area, it represents the dominant species among small mammals, with higher abundance in smaller patches (Mattos et al. 2021).

This marsupial is small‐bodied (18–43 g), solitary, nocturnal, and exhibits arboreal and scansorial habits (Faria et al. 2019). It feeds mostly on arthropods (mainly Hymenoptera, Isoptera, Hemiptera, and Coleoptera), small fruits, and occasionally birds. Also, it presents seasonal variation in its diet, with reproductive females feeding more heavily on insects during the mating season (Camargo et al. 2014). In the Cerrado, reproduction occurs seasonally, from the end of the cool‐dry season to mid/end of the warm‐wet season (Lopes and Leiner 2015).

### 2.3 Small Mammal Captures

In each of the 36 sampled patches we established a 200‐m trapping line located 30 m from patch edges to minimize edge effects. Each line consisted of 20 trap stations, each containing four live traps: two placed on the ground (one small Sherman and one Tomahawk) and two in the understory (one small and one large Sherman), totaling 80 traps per patch. Trapping occurred over the year 2018 between the rainy‐dry season (April–June) and the dry‐rainy one (August–October), accounting for seasonal variations in diet. Live trapping was carried out over four consecutive nights per field campaign, amounting to an effort of 640 trap‐nights per patch and 23,040 trap‐nights in total. Traps were baited with peanut butter, corn powder, sardine, and banana. Captured individuals were marked with a numbered ear tag, measured and weighed. Sex, reproductive status, and age were recorded. We collected a hair sample from the posterior dorsal region from all individuals with clean scissors and stored samples in plastic tubes. All procedures followed the guidelines of the American Society of Mammalogists for the use of wild animals in research (Sikes 2016). We conducted the study under the permission of the Committee of Ethics and Animal Use from the University of Brasília (28/2018), Instituto Chico Mendes de Biodiversidade (SISBIO 61990), and Secretaria de Meio Ambiente do Estado de Goiás (SECIMA/CEMan 006/2019).

### 2.4 Landscape Structure

Landscape types were classified using the 2016 land use and land cover map from the MapBiomas Project (collection 4.0), derived from Landsat imagery at 30‐m resolution. The temporal mismatch between the MapBiomas data and field campaigns is unlikely to be significant, as the study sites occur within a landscape with long‐established human occupation. Landscape selection was based on the proportion of five land cover classes: forest, savanna, agriculture, pasture, and mixed agriculture–pasture. Habitat loss was evaluated as the proportion of habitat amount available in each landscape context [10%, 25%, and 40% of natural cover (considering forest plus savanna)] (Figure 1). Patch areas were extracted (ha), ranging from 2 to 760 ha, and then were grouped into size classes on a log‐scale (Norris et al. 2010) to represent a potential gradient of fragmentation, as indicated in Table A1. From this point on, we will refer to patch size classes 1 (C1: 0–10 ha), 2 (C2: 11–100 ha), and 3 (C3: 101–1000 ha) to indicate the gradient of size representing small, medium, and large patches respectively.

### 2.5 Isotopic Analysis

We collected hair samples of at least three individuals of *Gracilinanus agilis* per patch, whenever possible (but we did not discard patches where we could only get one or two samples). We analyzed 98 samples from individuals captured in 30 of the 36 sampled patch sites (since in six patches we had no captures of *G. agilis* to collect samples). Sample size per patch size classes within landscapes ranged from six to 22 samples (Table A1). Also, we distributed samples among the sexes as equally as possible and excluded juveniles to avoid possible bias in isotopic values towards gender and age differences in trophic niche. Hair samples were washed with distilled water, immersed in a 2:1 solution of chloroform and methanol for 30 min, and washed again with distilled water. Afterwards, samples were oven‐dried for 12 h at 65°, shredded and weighed (minimum aliquot of 1.5 mg) in tin capsules on an analytical scale (0.001 g precision) (Ribeiro et al. 2019).

Isotopic analyses were performed at the Stable Isotope Facility of the University of California (SIF), Davis, USA. Samples were analyzed for ^13^C and ^15^N isotopes using a PDZ Europa ANCA‐GSL elemental analyzer interfaced with a PDZ Europa 20–20 isotope ratio mass spectrometer (Sercon Ltd., Cheshire, UK). During analysis, samples were interspersed with several replicates of at least four different laboratory reference materials. These reference materials have been previously calibrated against international reference materials, including: IAEA‐600, USGS‐40, USGS‐41, USGS‐42, USGS‐43, USGS‐61, USGS‐64, and USGS‐65 reference materials. A sample's provisional isotope ratio is measured relative to a reference gas peak analyzed with each sample. The standard used for carbon analysis was Vienna Pee Dee Belemite (Vienna PDB; ^13^C:^12^C ratio = 0.01118), and the standard used for nitrogen analysis was atmospheric air (^15^N:^14^N ratio = 0.0036765). These provisional values are finalized by correcting the values for the entire batch based on the known values of the included laboratory reference materials. The long‐term standard deviation is 0.2 per mil for ^13^C and 0.3 per mil for ^15^N.

### 2.6 Data Analysis

#### 2.6.1 Habitat Use and Trophic Position

To evaluate whether fragmentation and habitat loss lead the agile opossum to incorporate matrix resources (indicated by an increase in δ^13^C) and to test whether fragmentation causes a shift in trophic position (changes in δ^15^N values) depending on habitat loss, we compared nested linear mixed models (LMM) using a likelihood ratio test (LRT). We used LMM since residuals were normally distributed. To account for differences in the number of isotopic samples between patch sites and to avoid pseudoreplication, we included *site* as a random effect; the following variables—and the interaction between them—were included as fixed effects: (1) *Patch size class*, categorical variable representing fragmentation (following Table 1), with three levels (small, class 1: C1; medium, class 2: C2; large, class 3: C3); (2) *Habitat amount in the landscape* (%), categorical variable representing the gradient of habitat loss in the landscape, with three levels (landscape 10%, landscape 25%, landscape 40%). The significance of explanatory variables was given by changes in deviance and *p*‐values resulting from LRTs between all combinations of nested models built by dropping variables in a stepwise approach. We fit models using the *lme4* package (Bates et al. 2015), and performed model validation following Zuur et al. (2009) in R version 3.6.2 (R Core Team 2019).

**TABLE 1:** Summary of the linear mixed model analyses of the effects of habitat fragmentation and habitat loss on *Gracilinanus agilis* values of δ^13^C and δ^15^N hair contents.

	Variables	df	Deviance	χ^2^	*p*
δ^13^C	Patch size class: Landscape	4	212.890	4.879	0.770
	Patch size class + Landscape	2	214.840	1.947	0.745
	Patch size class	2	215.670	0.836	0.658
	Landscape	2	217.770	2.097	0.307
δ^15^N	Patch size class: Landscape	4	242.800	37.295	**1.016 × 10** ^**−5**^
	Patch size class + Landscape	2	265.300	22.503	**1.591 × 10** ^**−4**^
	Patch size class	2	275.420	10.111	**0.006**
	Landscape	2	272.690	7.385	**0.025**

*Note:* The significance of a variable was determined by changes in deviance and *p*‐values generated by comparing nested models that drop variables in a stepwise approach. Significant values are indicated in bold.

#### 2.6.2 Isotopic Niche Space

Isotopic niche metrics were based on mean values of δ^13^C—δ^15^N from *G. agilis* individuals dispersed in the isotopic niche space, represented by a biplot. These metrics reveal important aspects of trophic structure and are related to trophic diversity (Layman, Arrington, et al. 2007; Jackson et al. 2011). Therefore, to evaluate possible shifts in *G. agilis* trophic niche along the gradients of fragmentation and habitat loss, we calculated the standard ellipse areas corrected for small sample size (SEAc), a bivariate standard deviation that represents the core isotopic niche space of a population, which is calculated using 40% of the data to plot the ellipses (Jackson et al. 2011). For this, we pooled isotopic samples according to patch size classes (Table A1) nested within each landscape (10%, 25%, and 40%). Thus, we were able to compare the effects of patch size per se depending on the context of habitat loss. Also, we ensured we had at least five samples per size class/landscape to estimate SEAc (Jackson et al. 2011). We compared size and position between ellipses from patch size classes within landscapes. These analyses were conducted in R version 3.6.2 (R Core Team 2019) using the *SIBER* package (Jackson et al. 2011).

The raw data and code that generated all figures and tables are available as Mendeley Data, V2, doi: 10.17632/8v42zt7dyv.29 (Environmental Isotope Studies, EIS 2025).

## 3 Results

### 3.1 Habitat Use and Trophic Position

**FIGURE 2:**
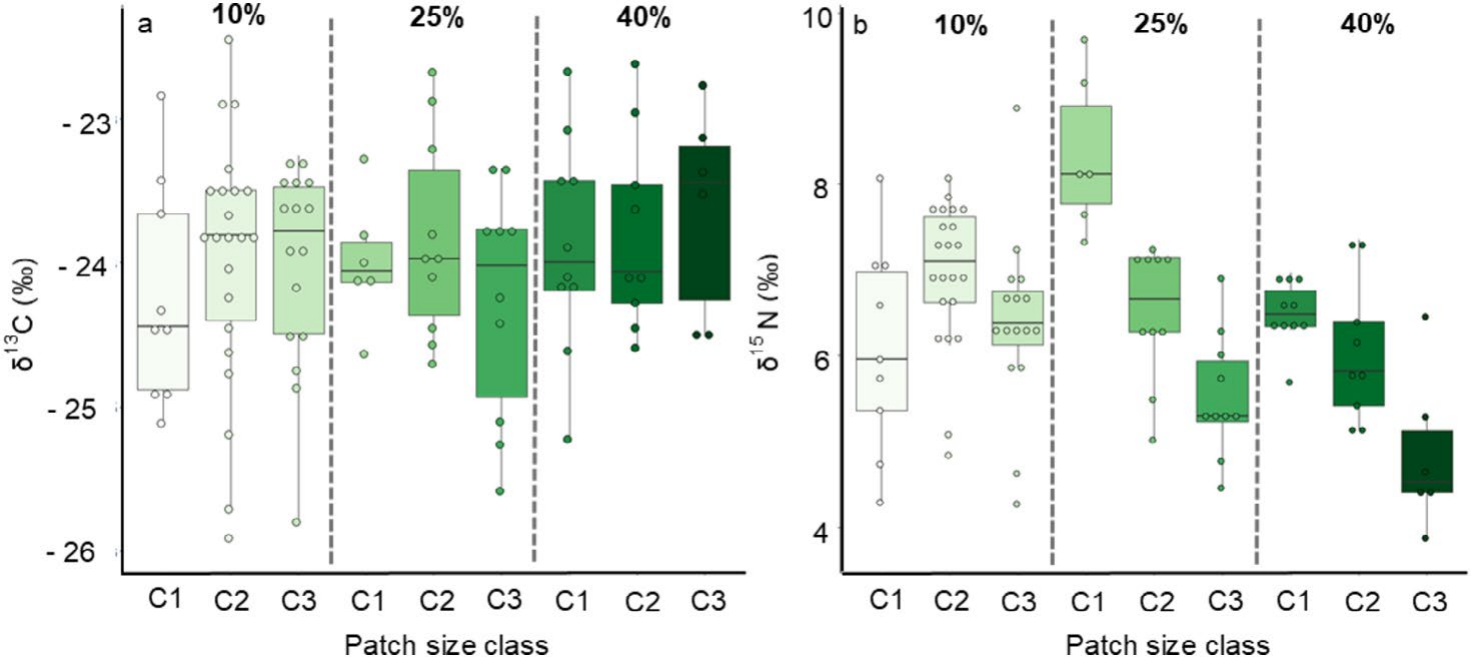
Distribution of (a) δ^13^C and (b) δ^15^N values from hair samples of *Gracilinanus agilis* across patch size classes within a landscape gradient of habitat amount (10%, 25%, and 40% natural cover) in semideciduous dry forests of the Brazilian Cerrado. The landscape gradient of remnant habitat amount is separated by dotted lines. Patch size classes (C1 – small; C2 – medium; C3 – large) within each landscape are represented by a green gradient, ranging from lighter green (more deforested) to darker green (more conserved). Each dot represents an individual.

Mean values of δ^13^C were similar across patch size classes within landscapes, ranging from −25.9% to −22.8‰ (Figure 2). This interval of δ^13^C values revealed that individuals assimilated mostly forest patch resources (C_3_) and practically no resources from the pasture matrix (C_4_), regardless of patch size and habitat loss context (Table 1, Figure 2). Patch size class and habitat loss in the landscape had no effect on δ^13^C values (Table 1, Patch size class, *p* = 0.658; Landscape, *p* = 0.307).

The agile opossum presented a great variation in δ^15^N values, ranging from 3.9‰ to 9.7‰ (Figure 1). These values indicate it is an omnivore‐insectivore with great trophic plasticity, presenting a diet that comprises frugivory up to third‐level consumer (Figure 2). The model that best explained variation in δ^15^N values included a significant interaction of patch size class with habitat loss in the landscape (Deviance = 242.8, χ^2^ = 37.295, df = 4, *p* = 1.016 × 10^−5^), which means the effects of fragmentation on δ^15^N values depended on the landscape context of habitat loss (Table 1, Table A2). In the most degraded landscape (10%), δ^15^N values were similar irrespective of patch size, ranging from 6.1‰ on average in small patches towards a 0.9‰ and 0.3‰ increase in medium and large patches, respectively (Table 1, Table A2, Figure 2). These values indicated that the agile opossum occupies approximately the same trophic level and feeds on similar food items (likely invertebrates) regardless of patch size. Moreover, in this extreme context of habitat loss, we found a similarly high range of δ^15^N values across all patch size classes, indicating a generalist feeding habit and a wide trophic niche width at the population level (Table 1, Table A2, Figure 2). However, in landscapes with intermediate (25%) and high (40%) habitat amount levels, we found a tendency to frugivory, revealed by the negative relation of δ^15^N values with patch size. Corroborating our hypothesis, this effect was stronger in the intermediate landscape (25%), with an estimated reduction of 2.7‰ in δ^15^N values from small (C1, 8.3‰) to large patches (C3, 5.6‰), corresponding to shifts of almost two trophic levels (Table 1, Table A2, Figure 2). The most conserved landscape (40%) showed a slightly declining pattern of δ^15^N values from small to large patches with an estimated reduction of 1.6‰. Also, δ^15^N values reached much lower levels in larger patches (C1, 6.5‰; C3, 4.9‰), indicating a more consistent contribution of basal resources (fruits and C_3_ leaves) when there is more habitat available at the landscape level (Table 1, Table A2, Figure 2).

### 3.2 Isotopic Niche Space

**FIGURE 3:**
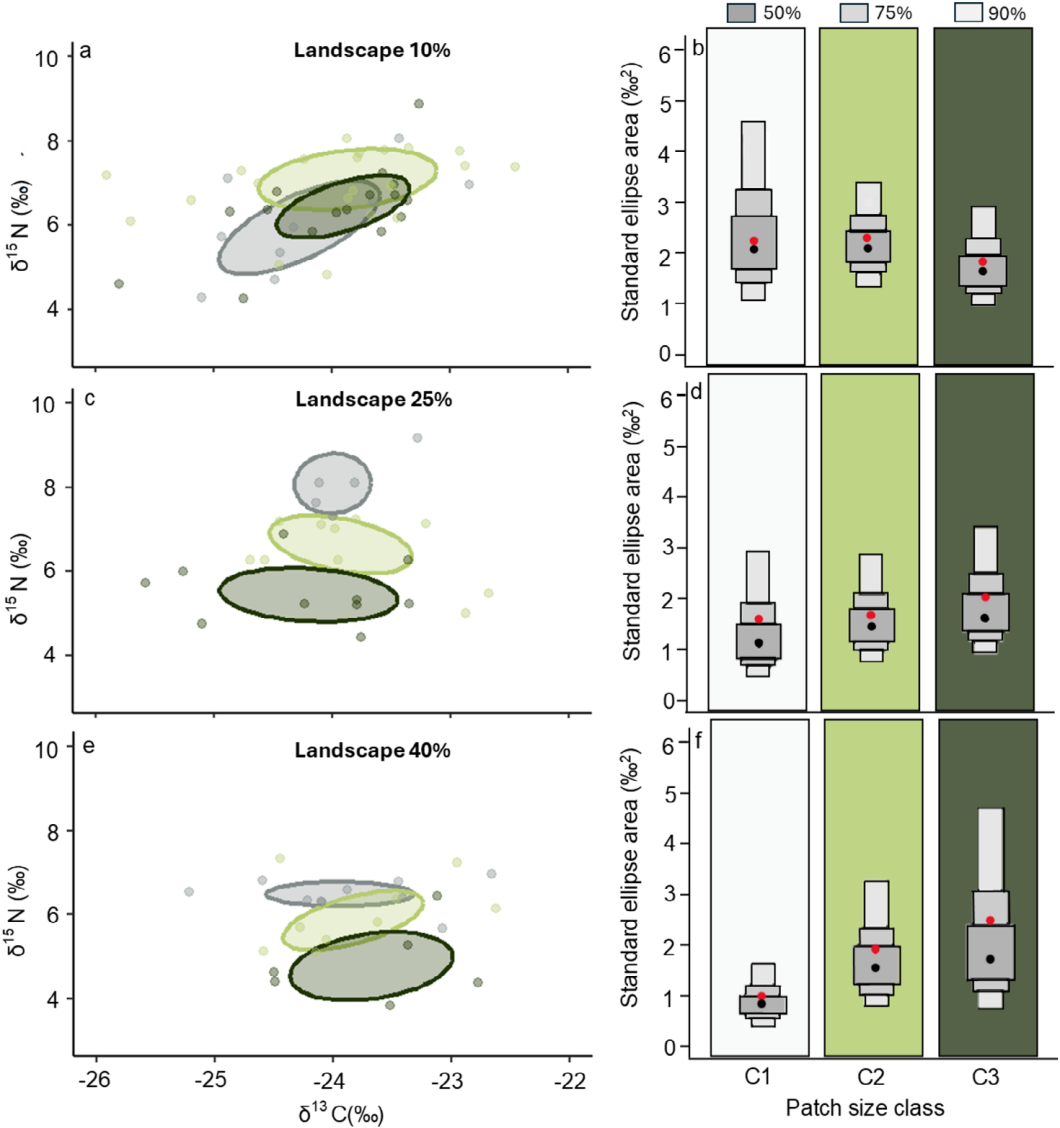
Isotopic niche space and standard ellipse area density plots of hair samples from *Gracilinanus agilis* across patch size classes along a landscape gradient of habitat amount in semideciduous dry forests of the Brazilian Cerrado. The landscapes represent different amounts of remaining habitat: (a, b) 10%; (c, d) 25%; and (e, f) 40%. Colors indicate patch size classes, ranging from small to large, as follows: Grey – class 1 (C1); light green – class 2 (C2); and dark green – class 3 (C3). Standard ellipse areas corrected for small sample sizes are estimated using Bayesian inference (40% of the data), and shaded rectangles above the density plots represent the 50%, 75%, and 95% credible intervals. Red dots in (b, d, f) represent standard ellipse areas corrected for small sample sizes (SEAc).

Isotopic niche ellipses varied among patch size classes along the landscape gradient of habitat loss (Figure 3), and overall shifts were associated with changes in δ^15^N values. In the most degraded landscape (10%), isotopic niche ellipses were similar in size and position among patch size classes (Table A3, Figure 3). However, the isotopic niche area (SEAc) from small patches (C1, 2.6‰^2^) was 1.4 times larger than in large patches (C3, 1.8‰^2^) (Table A3, Figure 3). In intermediate and more conserved landscapes, we found a progressive niche shift downward in the biplot niche space from small to large patch size classes, indicating a greater contribution of basal resources as patch size and habitat amount available in the landscape increase (Figure 3). We also observed a progressive expansion of niche area from small to large patches in both landscapes, with this expansion being more pronounced in the most conserved landscape (Landscape 25%: SEAc_C3_ was 1.3 times larger than SEAc_C1_; Landscape 40%: SEAc_C3_ was 2.6 times larger than SEAc_C1_, Table A3, Figure 3).

In summary, in the landscape with higher level of habitat loss (10% of habitat amount), despite the similarity of niche position across patch sizes, there was a slight expansion in niche area as patch sizes decreased. As habitat loss decreases in the landscape (landscapes with 25% and 40% of habitat amount), there was a progressive expansion of the ellipse areas (indicating greater trophic diversity) towards larger patches, along with a shift in trophic position from an omnivore‐insectivore diet to one more reliant on basal resources.

## 4 Discussion

Fragmentation effects on the isotopic niche space of *G. agilis* were dependent on the context of habitat loss in the landscape and were primarily linked to shifts in hair δ^15^N. We did not detect changes in δ^13^C values associated with fragmentation and/or habitat loss, but δ^13^C values indicated that the species is highly dependent on forest resources. In the most degraded landscape, the agile opossum exhibited a small expansion in niche area towards smaller patches, though there was no significant change in trophic position. At the intermediate level of habitat amount, there was a strong niche shift accompanied by a slight expansion in niche area from small to large patches. Lastly, in a more conserved landscape, a similar pattern emerged; however, the niche shift represented nearly one trophic level from small to large patches, with changes reflecting a much lower trophic position and a pronounced niche expansion. We suggest that different ecological mechanisms may underlie these changes, with their relative importance varying depending on the landscape context of habitat loss, leading to distinct responses in isotopic niche space.

Contrary to our predictions, we did not find fragmentation and/or habitat loss effects in δ^13^C values, revealing that *G. agilis* feeds on similar basal resources (primarily C_3_) across all study patches and is highly dependent on forest remnants. This suggests a potentially low tolerance to the pasture matrix from a trophic perspective (Magioli et al. 2019).

Our results also revealed that *G. agilis* presents high trophic plasticity in these altered landscapes and feeds on different trophic levels depending on patch size and landscape context of habitat loss. Contrary to our expectations, δ^15^N values were similar irrespective of patch size in the most degraded landscape, indicating no changes in trophic position. In degraded landscapes, patches are so isolated from each other that the species‐area relation is lost, since populations are exposed to low colonization rates and high extinction rates (Andren 1994; Pardini et al. 2010). If resource organisms (plants and arthropods) experience these effects, it could potentially lead to a homogeneous resource availability and diversity between patches of different sizes (Lôbo et al. 2011; Gámez‐Virués et al. 2015). Consequently, in an extremely simplified and degraded landscape, agile opossum individuals may remain spatially restricted within resident patches to forage on whichever resources are available within each patch, given its small home range and low dispersion ability (Ribeiro 2011; Shibuya et al. 2018). If resource biotas are homogenized across patches, it should cascade down through the food web (Wirth et al. 2007) and be reflected in the trophic position of generalist consumers (Layman, Quattrochi, et al. 2007).

For both the landscape with an intermediate level of habitat amount and the more conserved one, we found a negative relationship of patch size with δ^15^N values, corroborating our hypothesis. There was a general pattern of a gradual transition from a higher trophic level to a more basal consumer position from small to large patches. This effect was stronger in small patches in the intermediate landscape, where individuals occupied higher trophic positions nearly corresponding to a third‐order consumer. However, in larger patches within the more conserved landscape, individuals showed the lowest trophic level, revealing a higher contribution of basal resources to their diet.

Studies with several taxa across different ecosystems show that generalist consumers present shifts in trophic niche and occupy higher trophic positions under conditions of fragmentation or habitat alteration (Resasco et al. 2018; Magioli et al. 2019; Muñoz‐Lazo et al. 2019). We propose that shifts in trophic position may result from changes in the arthropod trophic structure with varying patch size and reduced fruit availability due to increased fragmentation and habitat loss. Fruit availability is lower in forest fragments compared to continuous forests (Terborgh 1986, 1992). If large patches (> 100 ha) are closer to pristine continuous forests in terms of habitat structure and plant diversity, they should also offer higher fruit availability compared to smaller patches, as observed in the study area (in prep.). Since fruits are a significant part of the diet of *G. agilis* (Camargo et al. 2014), it is expected that individuals will positively respond to higher availability of this basal resource, potentially resulting in a lower trophic position in larger patches, particularly in more conserved landscapes.

With lower fruit availability, *G. agilis* may increase its consumption of arthropod resources to meet energetic, nutritional, and water needs (Camargo et al. 2014). In intermediate landscapes, arthropod resources might be, therefore, more important to the opossum's diet than more basal resources, especially in smaller patches. Fragmentation can increase arthropod morphospecies richness at intermediate habitat amounts, likely due to enhanced resource diversity and favorable edge microclimates, consistent with the intermediate disturbance hypothesis (Crist and Ahern 1999; With and Pavuk 2011). However, arthropod guilds respond differently to fragmentation and habitat loss, with herbivores often negatively affected by habitat loss (Rossetti et al. 2014; Gámez‐Virués et al. 2015; Benítez‐Malvido et al. 2016), whereas omnivorous and specialized predators—and leaf‐cutting ants—may benefit from habitat loss or fragmentation (Wirth et al. 2007; Benítez‐Malvido et al. 2016; Gámez‐Virués et al. 2015). If shifts in the trophic structure of arthropod resource communities driven by fragmentation and habitat loss (With and Pavuk 2011) are reflected in the trophic position of a consumer across the gradient of habitat alteration (Layman, Quattrochi, et al. 2007; Resasco et al. 2018), then the observed changes in *G. agilis* trophic position in the intermediate landscape might result from synergistic effects of fragmentation and habitat loss on arthropod communities.

**FIGURE A1:**
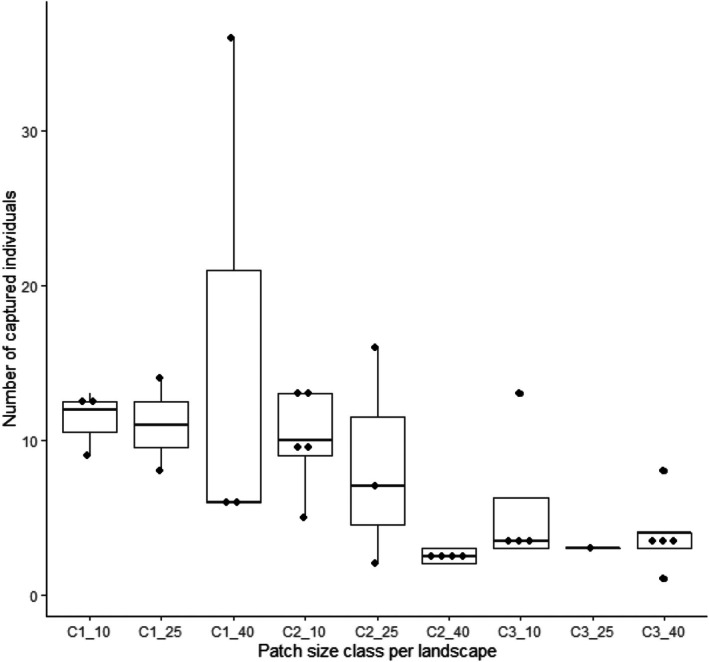
Number of captured individuals ( *Gracilinanus agilis* ) in each patch size class (C1 – class 1, C2 – class 2, C3 – class 3) within landscapes with different remnant habitat amount (10%, 25%, 40% of natural cover) in semideciduous dry forests in the Brazilian Cerrado.

In the most degraded landscape, *G. agilis* showed a slight niche expansion towards smaller patches, as predicted by our alternative hypothesis, but without shifts in the ellipse position. This suggests that the agile opossum feeds at the same trophic level regardless of patch size but incorporates a slightly higher diversity of food items in smaller patches. The higher trophic diversity in small patches compared to larger ones in this landscape may be explained by the effects of forager density on diet breadth. Under low resource availability, increased competition can cause a broader range of food items to be consumed (Emlen 1966; MacArthur and Pianka 1966; Schoener 1971), leading to a wider trophic niche (Roughgarden 1972; Fontaine et al. 2008). Indeed, in the study area, the agile opossum showed higher population density in small patches within the most degraded landscape [Figure A1; see Mattos et al. 2021], suggesting a high degree of intraspecific competition.

In the intermediate and more conserved landscapes, as predicted by our hypothesis, we observed a progressive niche shift from insectivory‐omnivory to frugivory‐omnivory, along with niche expansion from small to large patches. This shift was more pronounced in the more conserved landscape. In other words, increased fragmentation was associated with niche retraction. In these landscapes, forest basal resources (likely fruits) and primary consumer arthropod prey contributed progressively to the diversification of *G. agilis'* niche as patch size and habitat amount in the landscape increased. This pattern of niche shift may be linked to changes in forest structure and landscape connectivity resulting from fragmentation and habitat loss (Laurence et al. 2000), which can influence habitat use (Melo et al. 2013; Hannibal et al. 2018), resource exploitation (Klarner et al. 2017; Resasco et al. 2018), as well as resource diversity and availability (Laurence et al. 2000; Haddad et al. 2015).

Variations in microhabitat use lead to isotopic niche space differentiation in small mammals (Dammhahn et al. 2013; Galetti et al. 2016; Diniz‐Reis et al. 2025). Consequently, more complex forests with increased vertical stratification are expected to promote trophic niche partitioning. Large patches, particularly in more conserved landscapes, experience fewer edge effects and feature more complex, structured forests (Laurance et al. 2018), allowing for higher resource diversity—such as fruits and arthropods—to be exploited across different vertical strata (Benítez‐Malvido et al. 2016). This enhanced resource availability fosters spatial and dietary segregation among individuals, promoting specialization in specific food items and ultimately leading to an overall niche expansion at the population level (Camargo et al. 2019).

Our results indicated that habitat loss in the landscape dictates the strength of fragmentation effects on the niche responses of a small mammal in semideciduous dry forests in the Brazilian Cerrado. This supports predictions that overall habitat amount in human‐altered landscapes is a key predictor of biodiversity (Fahrig 2013; Melo et al. 2017; Hannibal et al. 2018). Furthermore, our findings demonstrate that the impacts of human activities on ecosystems extend far beyond species loss, influencing species' trophic ecology, niche breadth, trophic structure, and habitat use (Layman, Quattrochi, et al. 2007; Resasco et al. 2018; Magioli et al. 2019; Muñoz‐Lazo et al. 2019).

Isotopic analyses of generalist consumers provide a unique opportunity to better understand how key biotic and abiotic processes are altered by landscape fragmentation from a trophic perspective (Resasco et al. 2018), even though this approach does not capture other aspects of species ecology that may be critical in shaping responses to habitat alteration. Species that are tolerant to habitat modification, like the agile opossum, serve as good models for evaluating the effects of fragmentation across gradients of habitat modification on several ecological aspects (Layman, Quattrochi, et al. 2007; Resasco et al. 2018; Muñoz‐Lazo et al. 2019). Since these species may exist across the full environmental gradient, researchers can comprehensively document and investigate patterns of niche shifts, shedding light on trophic ecological changes driven by spatial effects or environmental alterations. In this sense, we observed niche shifts that could possibly indicate changes in the ecological role of the agile opossum across the fragmentation gradient, depending on the level of habitat loss. Landscape changes might reduce faunal fruit‐dispersal services, particularly in small patches, potentially limiting their ability to support forest regeneration in altered landscapes (Bregman et al. 2016; Bovo et al. 2018; Muñoz‐Lazo et al. 2019).

Moreover, we showed that even highly adaptable species like the agile opossum remain strongly dependent on forest resources, even in patches located within severely fragmented landscapes (< 10% habitat amount). Our study reinforces the importance of conserving forest patches of varying sizes, including small ones (< 10 ha), as they might provide refuges for forest‐dependent populations, sources of plant propagules, and stepping‐stones in human‐modified landscapes (Laurance et al. 2018). However, our results suggest that the ecological role of a species may be constrained in highly fragmented landscapes, with potential consequences for ecosystem functioning. Additionally, the consequences of fragmentation on habitat quality and its effects on consumer trophic ecology remain poorly understood. Therefore, we suggest that future studies investigate the direct impacts of changing environmental conditions within patches following fragmentation and habitat loss on trophic niche. Finally, seasonality has been shown to affect food availability in tropical ecosystems, influencing trophic niche variation in small flying and non‐flying mammals (Muñoz‐Lazo et al. 2019; Ribeiro et al. 2019; Camargo et al. 2021). Therefore, considering seasonal effects on trophic niche shifts may be important for better understanding how tropical species respond to landscape changes. In this context, our findings likely reflect patterns specific to semideciduous dry forests within the Cerrado biome in central Brazil and may not be directly applicable to other regions or biomes.

## Author Contributions

**Ingrid de Mattos:** conceptualization (equal), data curation (lead), formal analysis (lead), funding acquisition (lead), investigation (lead), methodology (equal), project administration (lead), resources (equal), visualization (lead), writing – original draft (lead), writing – review and editing (lead). **Juliana Fernandes Ribeiro:** conceptualization (equal), methodology (equal), validation (equal), writing – review and editing (equal). **Bárbara Zimbres:** conceptualization (equal), formal analysis (equal), methodology (equal), writing – review and editing (equal). **Gabriela Bielefeld Nardoto:** conceptualization (equal), methodology (equal), resources (equal), supervision (equal), writing – review and editing (equal). **Jader Marinho‐Filho:** conceptualization (equal), funding acquisition (supporting), resources (equal), supervision (equal), writing – review and editing (equal).

## Funding

This work was supported by Rufford Foundation, Rufford Small Grants 24992‐1. American Society of Mammalogists, Latin American Student Field Research Award 2019. Coordenação de Aperfeiçoamento de Pessoal de Nível Superior (CAPES). Decanato de Pesquisa e Pós‐Graduação da Universidade de Brasília (DPG‐UnB). Conselho Nacional de Desenvolvimento Científico e Tecnológico (CNPq). Fundação de Amparo à Pesquisa do Distrito Federal (FAPDF), FAPDF 1378/2016.

## Conflicts of Interest

The authors declare no conflicts of interest.

## Data Availability

The data and code that supported the findings of this study and that generated all figures and tables are available at https://data.mendeley.com/datasets/8v42zt7dyv/2.

## Appendix A

**TABLE A1:** Patch size and number of studied patches of semideciduous dry forests in the Brazilian Cerrado from each size class (C1: 0–10 ha; C2: 11–100 ha; C3: 101–1000 ha) over the landscape gradient of habitat amount (10, 25, 40% of natural cover), and total number of *Gracilinanus agilis* hair samples per patch size class and habitat amount used in the isotopic analysis.

Size class	Patch mean size ± SD (ha)	Patch size range (min‐max; ha)	Habitat amount (%)					
			10	25	40	10	25	40
			Number of patches			Number of isotopic samples		
C1	5.5 ± 2.3	2–10	3	2	3	9	6	10
C2	46.4 ± 26.8	13–91	5	3	4	22	9	10
C3	236.5 ± 207.4	121–760	4	3	3	16	10	6
		Total	12	8	10	47	25	26

**TABLE A2:** Estimated parameters and standard error (SE) for the relative effects of patch size and habitat loss (landscape habitat amount, 10%, 25%, and 40% of natural cover) on δ^15^N values of the agile opossum *Gracilinanus agilis* in semideciduous dry forests in the Brazilian Cerrado. Patch size classes: Small (class 1); medium (class 2); large (class 3).

Fixed effects	Parameter	Estimate	SE
Intercept	β0	6.0933	0.3393
Patch size class 2	β2	0.8858	0.4137
Patch size class 3	β3	0.3047	0.4344
Landscape 25%	β4	2.2550	0.5365
Landscape 40%	β5	0.3864	0.4724
Patch size class 2: Landscape 25%	β6	−2.6840	0.6778
Patch size class 3: Landscape 25%	β7	−3.0530	0.6966
Patch size class 2: Landscape 40%	β8	−1.2949	0.6207
Patch size class 3: Landscape 40%	β9	−1.9274	0.6746
Random effect			
Intercept	υ	0.1166	0.3415
Residual		0.6864	0.8285

**TABLE A3:** Standard ellipse area corrected for small samples (SEAc, ‰^2^) for the agile opossum ( *Gracilinanus agilis* ) according to patch size class within the landscape gradient of habitat amount (10%, 25%, and 40% of natural cover) in semideciduous dry forests in the Brazilian Cerrado. Patch size classes: Small (C1 – class 1); medium (C2 – class 2); large (C3 – class 3).

Ellipses	Habitat amount		
	10%	25%	40%
	SEAc (‰^2^)		
C1	2.6	1.6	1.0
C2	2.3	1.7	1.9
C3	1.8	2.0	2.5
