# Correction to “Habitat Loss Shapes Isotopic Niche Responses of a Didelphid Opossum to Fragmentation in Neotropical Semideciduous Dry Forests of Central Brazil”

**DOI:** 10.1002/ece3.74012

**Published:** 2026-07-20

Mattos, I. , J. F. Ribeiro , B. Zimbres , G. B. Nardoto , and J. Marinho‐Filho . 2026. “Habitat Loss Shapes Isotopic Niche Responses of a Didelphid Opossum to Fragmentation in Neotropical Semideciduous Dry Forests of Central Brazil.” Ecology and Evolution 16, no. 5: e73558. 10.1002/ece3.73558.42063657 PMC13127230

The name of the third author was incomplete and has been updated from “Gabriela Nardoto” to “Gabriela Bielefeld Nardoto”.

The online version of this article has been corrected accordingly.

We apologize for this error.

